# Evaluation of Dietary Habits, Type A Behavior Pattern and Its Relationship with Oral Health Status in Dental Undergraduate Students: A Cross-Sectional Study

**DOI:** 10.3390/jcm11061540

**Published:** 2022-03-11

**Authors:** Marta Olmos-Valverde, María Carrillo-Díaz, María José González-Olmo, Martín Romero-Maroto, Isabel Jiménez-Trujillo

**Affiliations:** 1 Department of Orthodontics, Rey Juan Carlos University, 28922 Alcorcón, Spain; marta.olmos.valverde@urjc.es (M.O.-V.); mariajose.gonzalez@urjc.es (M.J.G.-O.); martin.romero@urjc.es (M.R.-M.); 2 Department of Paediatric Dentistry, Rey Juan Carlos University, 28922 Alcorcón, Spain; 3 Department of Preventive Medicine and Public Health, Rey Juan Carlos University, 28922 Alcorcón, Spain; isabel.jimenez@urjc.es

**Keywords:** type A personality, binge-eating disorder, healthy lifestyle, oral health, body mass index, students, dental

## Abstract

Oral health status among dental students has been widely studied, and while the repercussions of certain factors, such as personality type, adherence to healthy lifestyle habits and certain eating patterns, have been considered in the past, this study aims to study the combination of such factors and to carry out, in addition, clinical examinations that could provide deeper knowledge of real oral health status. A sample of 195 dental students was gathered and basic sociodemographic data (gender, age, nationality, hygiene habits, body mass index (BMI)) were collected, and type A personality scale (ERCTA), emotional eating (EE) and healthy lifestyle scale (EVS) values were registered. Descriptive analysis, Pearson correlations, a hierarchical linear regression model and moderation analysis were performed. Results showed that higher EE values were associated with a higher BMI, an increase in the decayed, missing, and filled teeth (DMFT) index, a higher number of carious and filled teeth, a higher ERCTA and a lower adherence to the EVS. Likewise, a higher DMFT was associated with a higher BMI, higher bleeding on probing index (BOP) values, higher ERCTA values and lower adherence to EVS. Dental floss disuse, BMI, EE and EVS predicted 25.3% of DMFT. In addition, a type A personality has a moderating effect only in those with medium and high EE levels.

## 1. Introduction

The type A behavior pattern constitutes an important element in the study of university social environments/university populations, as it is characterized by a high level of self-demand and competitiveness. This pattern of behavior could be linked to unhealthy lifestyles in the absence of adequate coping strategies for dealing with stress and, as a consequence, could lead to obvious health risks, as described by Roseman and Friedman in the 1950s [1,2,3,4,5]. In fact, in today’s society, cardiovascular diseases and cancer are the main causes of mortality; both entities are considered to be disorders derived directly or indirectly from lifestyle patterns. This is why, for health care systems, lifestyle habits are a major concern [6,7]. The same is true for emotions, since inadequate management of emotions could contribute to a possible imbalance in healthy lifestyle habits and, more specifically, to altered eating habits, among other things. Previous research results indicate a marked tendency, in type A personality subjects, for a more impulsive and frequent intake of food [8], usually of poorer nutritional quality [9] and with high energy densities in response to negative emotions, with this pattern being known as emotional eating. Conversely, type B personality subjects are described as less prone to stress when they do not manage to achieve their goals [1,2,3,4,5,10,11]. Personality A subjects, therefore, tend to develop a pattern of ingestion with certain characteristics, such as urgency and overeating, in response to usually negative emotions, such as stress, anxiety, sadness or anger [12].

Both behaviors, type A personality and emotional eating behaviors, have been studied individually and jointly, and have been identified as possible factors involved in systematic diseases, such as obesity, anxiety, cardiovascular diseases, hypertension, etc… [13]. However, to date, there are no studies that associate these factors with oral health, especially in dental students.

The possible relationships among factors such as type A personality, emotional intake or high BMI could have a negative reflection (or impact) on oral health status, although it is true that the data reported by previous studies are focused on other psychological factors, such as depression and anxiety, rather than on personality types, despite being focused on a university population (medical students) [14].

Moreover, caries has a multifactorial etiology involving three main factors: the host (saliva and teeth), the microflora (plaque) and the substrate (diet); as well as a fourth factor: time. These are influenced by individual, family, and community factors, such as genetic and biological factors, the social environment, the physical environment, health behaviors and dental and medical care [15].

These caries risk factors have been widely described, but it could also be interesting to study other more specific variables and their interrelation in this sector of the population. Therefore, the objectives of this research are:

- To analyze the relationships among the variables under study, such as type A personality, adherence to a healthy lifestyle, emotional eating behavior, body mass index and oral health status in dental students.
- To determine whether emotional eating, poorer adherence to a healthy lifestyle, higher body mass index and poorer oral hygiene could exert a possible impact on DMFT.
- To investigate whether having a type A personality alters the effect of emotional eating pattern on oral health.

## 2. Materials and Methods

### 2.1. Study Design and Setting

A cross-sectional study was carried out from February to May 2021. All dental students at the Rey Juan Carlos University of Madrid (Spain) were invited to participate on a voluntary basis and without additional compensation. The ethics committee of the Universidad Rey Juan Carlos approved the study with registration number 2909201913219.

Before starting, the nature and purpose of the research was explained to all students, and they signed an informed consent form. To guarantee their anonymity, students were asked to identify themselves with the initials of their first and last names.

Students with any local or systemic condition (pathologies, disability and/or medication) that could affect their oral health status and those who did not provide informed consent were excluded from the study.

The sample size was calculated considering the number of dental students enrolled in the 2019/2020 and 2020/2021 academic years (N = 228), an acceptable margin of error of 5% and a confidence level of 95%, leading to an initial “N” of 144.

Before starting, two examiners were trained and calibrated by a specialist. The result of the kappa statistic during the calibration process was 0.91 (indicating good agreement).

The participants were divided into groups and were gathered in the waiting room of the Rey Juan Carlos university clinic. There, they were given instructions to complete the questionnaire and a member of the research team remained in the room to answer any possible doubts.

### 2.2. Measures

In the present study, data on basic sociodemographic aspects (gender and age), hygiene habits (brushing frequency on a scale of 1 to 6, 1 being never and 6 being 2 or more times per day, and daily floss use and use of fluorinated toothpaste, both formulated as dichotomous questions), body mass index (BMI), emotional eating behavior and type A behavior personality patterns were collected by means of a structured self-administered questionnaire. In addition, an oral examination was performed on all participants to record the DMFT index (sum of decayed teeth (CD), filled teeth (OD) and extracted teeth (ED)) [16] and the bleeding on probing index (BOP).

Emotional eating habits were assessed by means of the ten-item emotional eater questionnaire (EEQ) scale [17], with four Likert-type response options (1: never, 2: sometimes, 3: usually and 4: always). The final scores for this scale were calculated by summing each subject’s responses to the ten items. Subjects were classified into four groups according to the score obtained. Score between 0 and 5: non-emotional eater. Score between 6 and 10: low emotional eater. Score between 11 and 20: emotional eater. Score between 21 and 30: very emotional eater. Cronbach’s alpha reliability coefficient was 0.93.

To study the type A behavior pattern, the ERCTA scale [8] was used. The result of the Spanish validation ERCTA scale shows that the type A behavior pattern responds to two factors: stress, and ambition and self-demand at work. This scale is composed of 8 items, with 1 to 5 points for each question. An individual surveyed would present stress and self-demand work if an average score of 24 points were reached. Therefore, those individuals who exceed the value of 24 points were considered as subjects with a type A personality. Cronbach’s alpha for this scale was 0.94.

To evaluate whether the subjects have a healthy lifestyle, the EVS scale [18] was used. This consists of 12 items divided into 3 subscales (eating habits, smoking habits and rest habits). These items should be scored from 1 to 5 (1 totally disagree, 5 totally agree) and the sum of all the scores for each item will show whether the subject has a healthy lifestyle.

### 2.3. Statistical Analysis

Statistical analysis was performed with SPSS version 27 (SPSS Inc., Chicago, IL, USA). Data analysis included descriptive statistics of sociodemographic variables (mean ± standard deviation) and a Kolmogorov–Smirnov test to assess the assumption of normality, which was confirmed. Descriptive analyses and Pearson correlations were performed on the variables of oral health, eating habits, type A personality and lifestyles, and hierarchical linear regression models were used to search for predictors of DMFT. Subsequently, to investigate whether personality type A had moderating effects on the relationship between DMFT and EE, a simple moderation analysis (model 1) was performed using the Hayes PROCESS module (version 3.3) for SPSS version 27, (SPSS Inc., Chicago, IL, USA). Cronbach’s alpha was also calculated to assess the internal consistency of the instruments.

## 3. Results

### 3.1. Sociodemographic Characteristics

The sample consisted of 195 subjects (50 males and 145 females), with a mean age of 24.5 ± 5.7 years old, a BMI of 22.2 ± 3.5, DMFT of 4.5 ± 4.6, a mean number of teeth with caries of 1 ± 1.7, a mean number of filled teeth of 3.2 ± 3.6, a mean number of extracted teeth of 0.2 ± 0.6 and a bleeding on probing index (BOP) value of 0.65%. Gingival health (bleeding less than 10%) was present in 92.3% of the sample. Regarding daily dental hygiene, 92.3% of the sample brushed their teeth more than twice a day, the use of fluoride paste was 92.8%, while only 62.6% used dental floss. The means and standard deviation found for EE was 10.8 ± 6.4, for ERCTA was 26 ± 3.7 and for EVS was 38.2 ± 8. Significant differences were found between genders in the ERCTA scale (women: M = 26.4 ± 3.4; men: M = 24.8 ± 4.3; t = 2.6; *p* < 0.01), with the women group showing higher scores. No significant differences were found for the rest of the variables.

### 3.2. Relationships among the Variables of Oral Health, Eating Habits, Type A Personality and Lifestyle

The relationships among the variables DMFT, BOP, EE, ERCTA and adherence to the EVS were analyzed.

As can be seen in Table 1, it was observed that a higher EE was associated with a higher BMI, an increase in the DMFT index, a higher number of decayed and filled teeth, a higher ERCTA and lower adherence to the EVS. Additionally, a higher DMFT was associated with a higher BMI, a higher BOP, ERCTA (although this is not statistically significant) and a lower adherence to EVS.

### 3.3. Predictor Variables of DMFT

A hierarchical multiple regression was performed to determine whether the analysis of the set variables of floss disuse, BMI, EE and EVS significantly predicted DMFT. The full model of flossing, BMI, EE and EVS (Model 4) was statistically significant R2 = 0.268, F(1, 189) = 17.313, *p* < 0.001; adjusted R2 = 0.253. See Table 2 for full details on each regression model.

### 3.4. Moderation Analysis of Personality Type on EE and DMFT

The sample was divided into two groups according to whether they presented type A personality or not: (Yes for ERCTA ≥ 25; No for ERCTA < 25). A moderation analysis was conducted with EE levels as an independent variable, DMFT as a dependent variable and personality type A as a moderating variable. The regression analysis, in which EE levels were considered to be a predictor of DMFT, had a *p* = 0.40, and the same occurs for the moderator variable (*p* = 0.59); however, a significant value was obtained for the interaction between the independent and moderator variable (*p* = 0.04; SE = 0.11; (0.01, 0.42)). Obtaining a significant value for this interaction indicates the presence of a moderator effect, which suggests that a type A personality interferes with the effect of emotional eating on DMFT.

**Figure 1:**
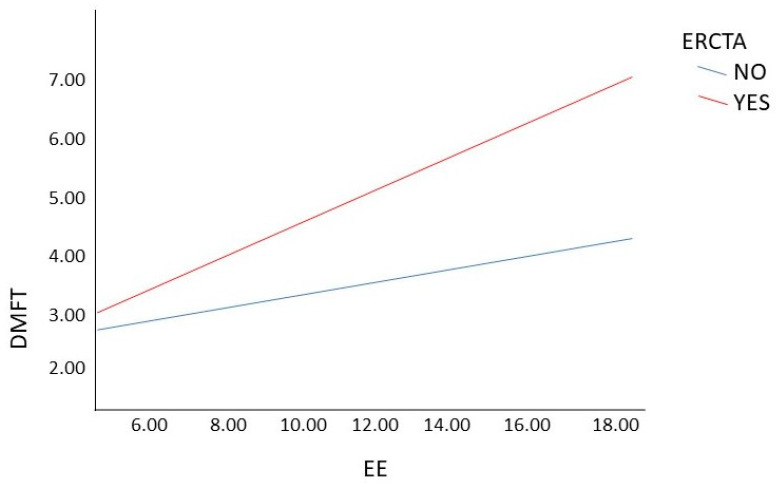
Moderation analysis of personality type A on emotional eating and DMFT. Note: DMFT = index to determine decayed, missing and filled teeth; ERCTA = core on type A personality scale; EE= emotional eating.

To determine when personality type A had a moderating effect in the main model, we analyzed the significance level and the upper and lower limits. In this case, we observed that emotional eating had a moderating effect only in those with medium EE levels (*p* = 0.013; SE = 0.67; (0.35, 3.03)) and even more so in those with high EE levels (*p* = 0.003; SE = 0.101; (1.08, 5.09)). However, it had no effect on those with low EE levels (*p* = 0.741; SE = 0.89; (−1.47, 2.06)) (Figure 1). In summary, we observed that Type A personality interfered with the effect of EE on the DMFT index.

## 4. Discussion

The results of the study highlight the possible relationship between type A personality variables, the tendency to perform emotional ingestions and actual clinically quantified oral health status, results which are supported by previous investigations described in the literature [14,19,20,21,22]. Furthermore, this study explored the role of body mass index, flossing, healthy lifestyle habits (in terms of balanced diet, smoking and sleep) and emotional eating in relation to oral health status (DMFT). The results have shown that individuals with high values in type A personality tests have a greater tendency to engage in emotional eating and to show higher BMI values. The relationship between emotional eating and high BMI has been suggested in previous studies, such as that of Van Strien et al. [20], and although they analyzed individuals with depression, the mediating factor for the increase in BMI was emotional eating. Likewise, Jonnes et al. [15] showed the relationship between maladaptive mechanisms to stress, such as emotional eating and smoking, and high BMI values. To date, the mediating role of stress reactivity to cortisol in the relationship between obesity and certain negative psychological variables, such as anxiety or depression, is not clear [23,24].

Regarding the relationship observed in the present study between emotional eating and higher DMFT values, there are no specific studies in the literature on this subject, but there are some that relate impulsive eating patterns associated with incorrect management of negative emotions and poorer dietary quality of intakes [25]. In addition, lower salivary flow values have been detected in patients with poor responses to stress, and these low saliva flows are associated with increased susceptibility to caries [26].

Regarding the relationship between DMFT and high BMI, there are previous studies on this subject that support our results confirming this association [27]. Both variables are related to the type of diet, in such a way that a higher intake of sugars can result in weight gain and also in a poorer oral health status. Sanders et al. [28] verified the well-known relationship between a higher intake of sugars, sugar-sweetened beverages and fruit juices and the presence of a greater number of carious lesions. On the other hand, a diet rich in vegetables, whole grains and omega-3 are associated with fewer caries. For these authors, DMFT is associated with a higher BMI.

Our research results show that unhealthy lifestyle habits are associated with poorer gingival health. Smoking and an unbalanced diet have been described as risk factors for severe gingivitis [29]. A diet rich in sugars increases the subgingival temperature and the emigration of neutrophils to the crevicular fluid, which, combined with the harmful effects of tobacco, can favor the development of this pathology. On the other hand, abundant periodontopathogens belonging to the group known as the “red bacterial complex” were found in subjects whose diet was rich in vegetables, fish and fruit, so it could be considered to be a protective factor against periodontitis [30]. Likewise, a correlation has been described between smoking and a higher consumption of carbonated beverages with high sugar content, and although these variables have not been specifically recorded in this study, they could explain why the results associate an unhealthy lifestyle with an increase in DMFT [31].

Previous research has described that there is a negative association between the consumption of red meat, processed meats and sweets and the onset of negative emotions, such as anxiety and emotional stress, contributing to the development of emotional eating [25].

This study’s results suggest that dental students who present a type A personality should be considered to be a risk group with regard to their eating habits because of their possible impact on systemic health and oral health.

Mindfulness meditation can be a useful tool in the management of negative emotions, such as irritability or anxiety [13] (with mindfulness usually deficient in individuals with type A personality) [21], and which are also predecessors of emotional eating. In addition, a multidisciplinary approach between psychologists, physicians and nutritionists is recommended, since dietary and lifestyle habits are modifiable [32].

In addition, previous studies have emphasized that early childhood is the appropriate time to promote oral health care protection habits, which allows for the future prevention of diseases such as dental caries and gingivitis. Therefore, it would be interesting to carry out preventive programs aimed at school-aged children to promote adequate hygiene and eating habits that will contribute to the improvement of general and oral health [33]. It is also important to point out some of the limitations of this study. First, the representativeness of the convenience sample could be questioned. In addition, the age of the participants was within the range or mean of similar studies [14,34,35]. With regard to gender, there was not an equal distribution, since female enrollment is usually higher in most health-related careers in our environment. In addition, the average DMFT value obtained in the research was within the range found in previous oral health surveys conducted among dental students [36]. Another limitation comes from the use of self-report measures, which may be affected by recall bias and responses based on social desirability. On the other hand, to diagnose caries and to confirm the presence of fillings, bitewing radiographs were not performed, so biases may exist in this regard. Furthermore, although a lot of the variables that may contribute to DMFT values have been taken into account for the study, such as hygiene habits (frequency of brushing, flossing, and the amount of fluoride in dental products), dental check-ups, smoking, sleep, diet and other psychosocial variables (such as type A behavior pattern and nutrition from an emotional perspective), other possible factors such as medication, chronic diseases, salivary flow, microorganisms, dental anatomy, xylitol intake, etc., have not been taken into account. Furthermore, non-carious lesions data have not been collected, even when they are highly prevalent, and its relation to dietary habits, psychological factors and their repercussions in oral health status are well known [37]. Moreover, the cross-sectional design of the study makes it impossible to adequately capture the nature of the dynamic relationships that may exist among the study variables, and causal relationships cannot be established. It would be particularly useful to open new lines of research and to perform a longitudinal analysis to clarify the relationships between such variables, and especially to identify how oral health status may be altered as a consequence of emotional eating habits and type A personality, since both factors share triggers such as poor stress management, tendencies to possess psychological patterns such as alexithymia and altered management of negative emotions [11,36].

## 5. Conclusions

Findings suggest that among dental students with higher type A personality values, high emotional eating and low adherence to a healthy lifestyle are related to worse oral health. In addition, a type A personality is a moderating factor in the relationship between emotional eating and DMFT. 

## Figures and Tables

**Table 1:** Associations among variables of oral health, eating habits, type A personality and lifestyle.

	BMI	DMFT	D	F	M	BOP	EE	ERCTA	EVS
BMI	1								
DMFT	0.333 **	1							
C	0.251 **	0.603 **	1						
F	0.253 **	0.912 **	0.249 **	1					
M	0.265 **	0.330 **	0.216 **	0.150 *	1				
BOP	0.102	0.193 **	-0.052	0.267 **	−0.037	1			
EE	0.347 **	0.319 **	0.325 **	0.216 **	0.130	−0.098	1		
ERCTA	0.143 *	0.130	0.020	0.152 *	−0.016	−0.032	0.149 *	1	
EVS	−0.161 *	−0.339 **	−0.145 *	−0.319 *	−0.173 *	−0.201 **	−0.334 **	−0.234 **	1

Note: DMFT = index to determine decayed, missing and filled teeth; D = number of decayed teeth, M = number of missing teeth, F = number of filled teeth; BOP = bleeding on probing index; EE = score on emotional eating scale; ERCTA = score on type A personality scale; EVS = healthy lifestyle score; * significant at the 0.05 level; ** significant at the 0.01 level.

**Table 2:** Results of the regression analysis to predict DMFT based on BMI, dental floss disuse, EE and EVS.

	DMFT							
	Model 1		Model 2		Model 3		Model 4	
Variable	B	β	B	β	B	β	B	β
Constant	−5.1 *		−1.2		−0.9		4.4 *	
BMI	0.4 **	0.3	0.4 **	0.3	0.3 **	0.2	0.3 **	0.2
Dental floss disuse			−2.6 **	−0.2	−2.4 **	−0.2	−2.2 **	−0.2
EE					0.1 **	0.2	0.1 **	0.2
EVS							−0.1 **	−0.2
R^2^	0.109		0.184		0.224		0.268	
F	23.5 **		21.5 **		18.3 **		17.3 **	
ΔR^2^	0.109		0.075		0.040		0.044	
ΔF	23.5 **		17.6 **		9.8 **		11.2 **	

Note. N = 195, * *p* < 0.05, ** *p* < 0.01.

## Data Availability

The data that support the findings of this study are available on request from the corresponding author. The data are not publicly available due to privacy and ethical restrictions.
